# A potential bioactive hard‐stock fat replacer comprised of a molecular gel

**DOI:** 10.1002/fsn3.433

**Published:** 2016-10-23

**Authors:** Michael A. Rogers, Paul A. Spagnuolo, Tzu‐Min Wang, Leonard Angka

**Affiliations:** ^1^ Department of Food ScienceUniversity of GuelphGuelphONCanada; ^2^ Department of Food ScienceRutgers UniversityNew BrunswickNJUSA

**Keywords:** apoptosis, ceramides, oleogel, organogel, sphingolipids

## Abstract

Short‐chain ceramides, such as *N*‐acetoyl‐d‐erythro‐sphingosine (C2), have a remarkable ability to structure edible oils, such as canola oil, into self‐standing organogels without any added saturated or *trans* fats. These short‐chain ceramides are ubiquitously found in foods ranging from eggs to soybeans. As the ceramide fatty acid chain length increases, there is an increase in the melting temperature of the organogel and a decrease in the elastic modulus. Gelation ability is lost at 2 wt% when the fatty acid chain length increases to six carbons; however, organogels form at 5 wt% up to 18 carbons. Short‐chain ceramides, C2, decrease cell viability of colon, prostate, ovarian, and leukemia cell lines, while ceramides with long‐chain fatty acids, C18, do not affect the viability of these cancer cell lines. This suggests that a bioactive spreadable fat, with no *trans* or added saturated fat, with the potential to alter the viability of cancer cell growth, is possible.

## 1 Introduction

Noncommunicable diseases, often related to diet, are now responsible for a larger percentage (46.8%) of the mortality rate than communicable disease (41.0%) (Shetty & Schmidhuber, 2011), leading global health leaders to shift attention from germs to what United Nations Secretary General Ban Ki‐Moon calls “a public health emergency in slow motion” (Zafar, 2011). As such, concerns related to diets elevated in *trans* fats due to their detrimental consequences ranging from unfavorable effects on lipoprotein (cholesterol) profiles, increased prevalence of heart disease, and metabolic syndrome have been raised. Governments have responded by removing the generally recognized as safe (GRAS) status for *trans* fats and are passing legislation to limit, and in some cases, are banning the application of *trans* fats (FDA, 2015). Removal of GRAS status for *trans* fats necessitates the substitution of certain hard‐stock fats, including partly hydrogenated fats. Unfortunately, the only current alternative to structuring with *trans* fats is substituting them with saturated fats, which are often negatively viewed by the consumer. Hence, alternatives to traditional triacylglyceride (TAG) structuring must be vigorously pursued.

Organogels comprising “small” molecular gelators (i.e., molecular gels) are thermally reversible, quasi‐solid materials comprised mainly of organic liquids that undergo spontaneous formation into self‐assembled networks that are often fibrillar in nature (SAFiNs) (George & Weiss, 2006; Mallia, Butler, Sarkar, Holman, & Weiss, 2011; Weiss & Terech, 2006). The contrasting, noncovalent, gelator–gelator and gelator–solvent interactions result in fibrillar aggregates that are, in some cases, capable of structuring fluids, preventing flow, and improving the mechanical properties of solids (Fahrländer, Fuchs, Mülhaupt, & Friedrich, 2003; Isare et al., 2009; Wilder, Hall, Khan, & Spontak, 2003). Specifically, organogels are being widely studied in structuring edible oils to utilize them as hard‐stock fat replacers (Bot & Agterof, 2006; Bot, den Adel, & Roijers, 2008; Bot, Veldhuizen, den Adel, & Roijers, 2009; Co & Marangoni, 2012; Da Pieve, Calligaris, Co, Nicoli, & Marangoni, 2010; Marangoni, 2013; Pernetti, van Malssen, Floter, & Bot, 2007). The ability of molecules to spontaneously self‐assemble into supramolecular, one‐dimensional (1D) aggregates requires an intricate balance between contrasting enthalpic and entropic parameters, including solubility, and those that control crystal growth. The 1D fibers, tubules, or ribbons (Terech, Rodriguez, Barnes, & McKenna, 1994) further interact forming a 3D bicontinuous network, held together by weak noncovalent interactions in a similar fashion to colloidal fat crystal networks (Suzuki et al., 2003). The importance of the supramolecular structure has been shown to be responsible for macroscopic entrapment of the liquid edible oil (i.e., gelation) via capillary forces and interfacial tension within the pores of the network (Li & Liu, 2010; Terech & Weiss, 1997).

With so many classes of known molecular gelators, why focus on ceramides? What is truly remarkable about this system, beyond there to structure liquid oils, is that ceramides have numerous beneficial health effects. Ceramides, often referred to as tumor suppressing lipids (Parodi, 2001), promotes TNF‐α, produced and excreted in the white blood cells and in the endothelium, and interleukin 1B that both led to apoptosis in tumor cells (Cremesti & Fischl, 2000). There are numerous potential health implications for the development of a ceramide‐based, spreadable fat product. Possibly, most exciting is the role of ceramides as inhibitors of colon carcinogenesis (Schmelz & Merrill, 1998a). Since ceramides alter cell growth, differentiation, and programmed cell death (i.e., apoptosis) (Schmelz, 2000), they may exert their effect on developing malignant adenocarcinomas located in the colon or elsewhere. Herein, we examine the role of ceramide fatty acid chain length on the physical properties of their edible organogels and on their ability to induce cell death of various cancer cell lines.

## 2 Materials and Methods

**Figure 1:**
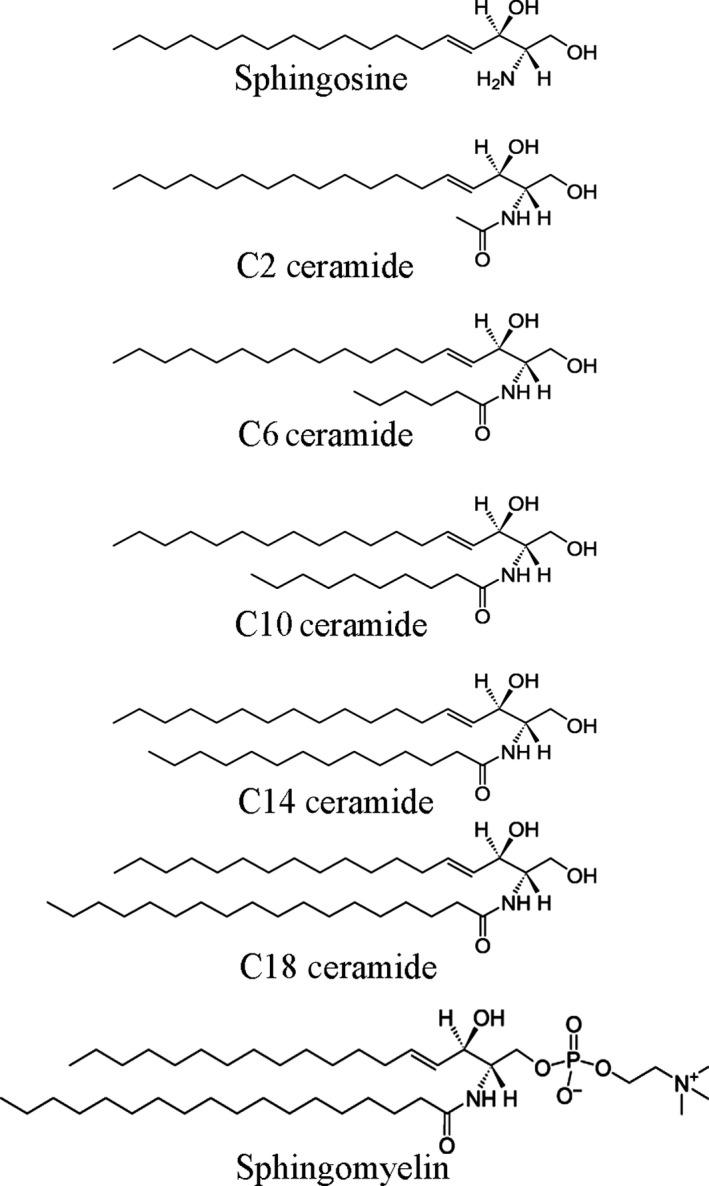
Chemical structure of sphingolipids

Different chain length ceramides (*N*‐acetyl‐d‐erythro‐sphingosine [C2 ceramide], *N*‐hexanoyl‐d‐erythro‐sphingosine [C6 ceramide], *N*‐decanoyl‐d‐erythro‐sphingosine [C10 ceramide], *N*‐myristoyl‐d‐erythro‐sphingosine [C14 ceramide], and *N*‐stearoyl‐d‐erythro‐sphingosine [C18 ceramide]), d‐erythro‐sphingosine (sphingosine), and *N*‐stearoyl‐d‐erythro‐sphingosylphosphorylcholine (sphingomyelin) were obtained from Avanti Polar Lipids (Alabaster, AL, USA) with a minimum purity of 99% (Figure 1). The ability of different chain length ceramides to gel edible oil was assessed at 2 and 5 wt% in canola oil (ConAgra Foods, NE). After being mixed in their appropriate ratios, samples were heated to 90°C for 5 min and then cooled to 20°C and stored for 24 hr before further analysis.

### 2.1 Differential scanning calorimetry

Five to 8 mg of 2 or 5 wt% sample was placed into aluminum pans and hermetic lids were sealed and placed into Q2000 DSC (TA instruments, New Castle, DE, USA), where the cell was constantly flushed with N_2_ gas. A three‐point calibration was carried out using empty pans, sapphires, and indium. The initial heating cycle was discarded because the thermal history was not controlled of the initial sample. The sample was heated to 95°C at 5°C/min and maintained for 5°C min to erase the crystal history, then cooled to 15°C at 5°C/min to observe the crystallization profile, and then reheated to 95°C to measure the melting profile. The thermal properties were measured in triplicate.

### 2.2 Small deformation oscillatory rheology

A discovery H2 Rheometer (TA Instruments, New Castle, DE, USA), equipped with an 8‐mm cross‐hatched flat plate geometry, was used to perform oscillatory stress and frequency sweeps in triplicate. The sample was placed on the temperature controlled Peltier plate and the 8‐mm diameter cylindrical geometry was lowered and the sample was compressed from the original height of 3000 to 2700 μm to ensure sufficient contact between the sample and spindle. There were two procedures for each gel sample: a frequency sweep using an oscillation frequency from 0.1 to 50 Hz at 1 Pa, followed by a second procedure using an amplitude sweep between 0.1 Pa and 50 Pa, with a frequency of 1 Hz.

### 2.3 Cell Culture

Unless otherwise stated, all cells were cultured in media supplemented with 10% fetal calf serum (FCS; Hyclone, Logan, UT) and antibiotics (100 units/ml of streptomycin and 100 μg/ml of penicillin; Sigma Chemical). The acute (AML) and chronic myeloid leukemia (CML) cell lines (OCI‐AML2, KG1a, U937, K562, TEX, and HL60 cells) were cultured in Iscove's modified Dulbecco's medium (IMDM; Life Technologies; Grand Island, NY). TEX leukemia cells were cultured in IMDM supplemented with 15% FCS, antibiotics, 20 ng/ml stem cell factor, 2 ng/ml IL3 (Peprotech; Hamburg Germany), and 2 mmol/L l‐glutamine (Sigma Chemical). Prostate cancer cell line, DU145; human myeloma cell line, LP1; ovarian cancer cell line, HeLa; and colon cancer cell lines, HCT116, HT29, COLO 205, and DDL‐1 were cultured in RPMI (Life Technologies). Cells were incubated in triplicate in a humidified air atmosphere containing 5% CO_2_ at 37°C.

### 2.4 Cell growth and viability

The effect of sphingolipids on cancer cell viability was assessed as described previously (Angka et al., 2014; Lee et al., 2015). Briefly, cell lines (1.5×10^4^/well) were seeded in 96‐well polystyrene tissue culture plates. After seeding, cells were treated with increasing concentrations of individual sphingolipids with a final DMSO concentration of less than 0.05%. The stock solution (5 mmol/L) was diluted in phosphate buffered saline (PBS), aliquoted and stored at −20°C to prevent excessive freeze thaw cycles. Cell growth and viability was measured using the 3‐(4,5‐dimethylthiazol‐2‐yl)‐5‐(3‐carboxymethoxyphenyl)‐2‐(4‐sulfophenyl)‐2H‐tetrazolium inner salt (MTS) reduction assay (Promega, Madison, WI) after 72 hr according to the manufacturer's protocol. Optical density was measured at 490 nm and cell growth is expressed as percent viable compared to vehicle control treated cells.

### 2.5 Statistical analysis

Unless otherwise stated, the results are presented as mean ± SD. Data were analyzed using GraphPad Prism 4.0 (GraphPad Software, USA). *p* ≤ .05 was accepted as being statistically significant.

## 3 Discussion

**Figure 2:**
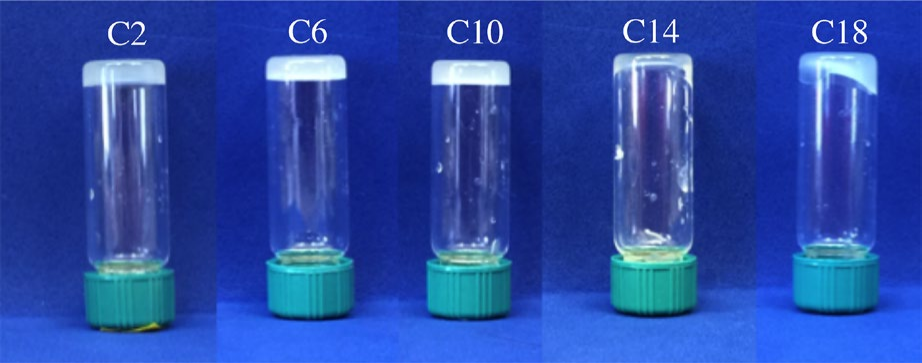
Visual appearance of different chain length ceramides at 2 wt% in canola oil inverted for 1 hr

The ability of sphingolipids to self‐assemble into a continuous 3D network capable of structuring edible oils is highly dependent on their chemical structure. After 24 hr at 20°C, all of the ceramides tested were able to gel canola oil at 5 wt% (data not show), while at 2 wt% only C2, C6, and C10 immobilized the oil sufficiently to prevent flow when the vial was inverted (Figure 2). As the chain length of the ceramide increased, so did the turbidity of the gel indicating that there was a coarsening of the supramolecular network of the gel or a change in the microstructural elements capable of diffracting light (Rogers, Wright, & Marangoni, 2008, 2009a). This was also previously reported where C2 ceramides produced a translucent gel and egg‐derived ceramide, comprised of mainly long‐chain ceramides, produced an opaque viscous solution (Rogers, Wright, & Marangoni, 2009b).

**Figure 3:**
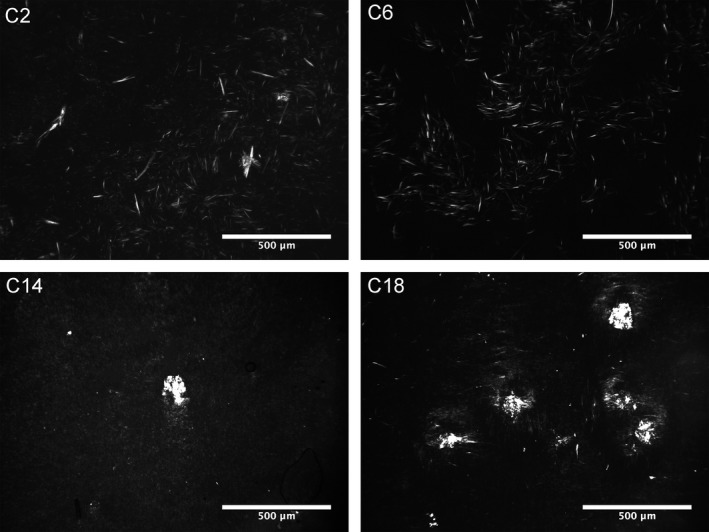
Polarized light micrographs of 5 wt% C2, C6, C14, and C18 in canola oil after being stored for 24 hr at 20°C. Mean storage (G′)(a) and loss modulus (G″)(b) at 5 wt% obtained from the frequency sweep. Asterisk indicate statistical significance at p < .05.

To visualize the changes in the microstructural elements and supramolecular network established by ceramides, polarized light microscopy was used (Figure 3). Under polarized light, it is clear that ceramides capable of limiting flow at 2 wt assembled into fibrillar crystals; samples that exhibited flow at 2 wt% assembled into sphereulitic crystals (i.e., C14 and C18 in Figure 3). It is also evident from the micrographs that the Sphereulitic are much larger and impede light from passing through the sample causing them to be opaque (Figure 2).

**Figure 4:**
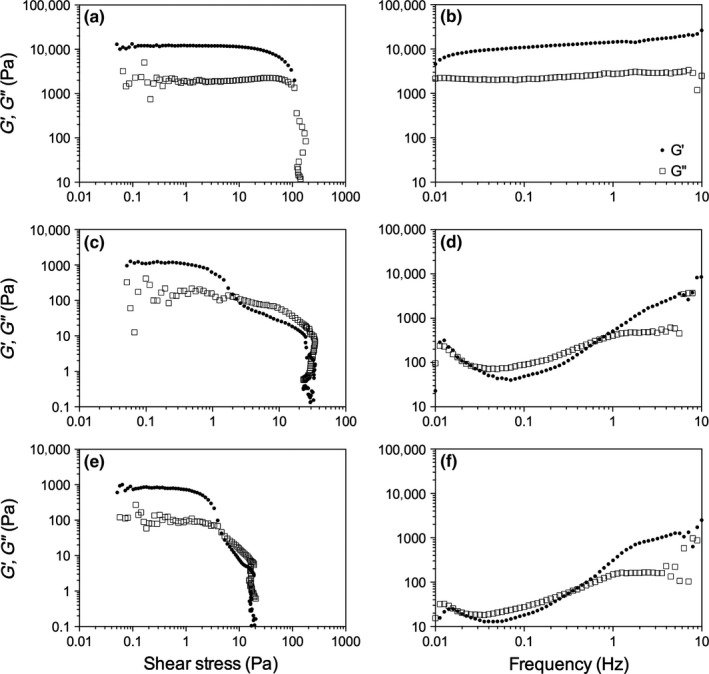
Small deformation oscillatory shear stress (a, c, e) and frequency sweeps (b, d, f) for 2 wt% C2 (a, b), C6 (c, d), and C10 (e, f)

**Figure 5:**
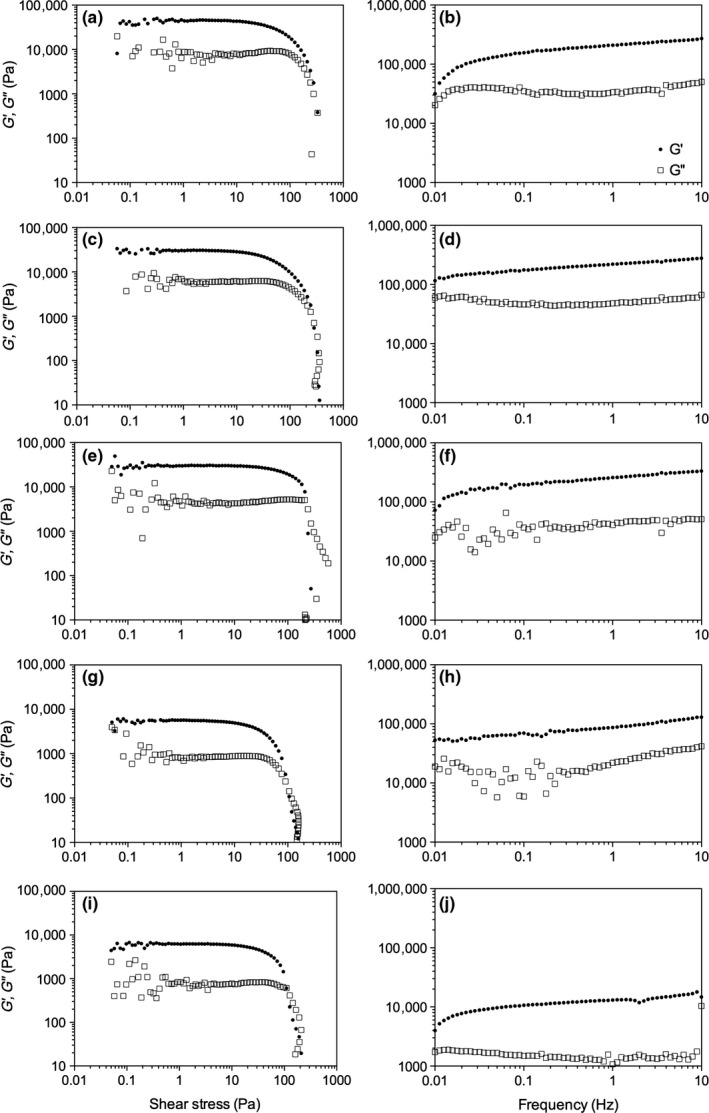
Small deformation oscillatory shear stress (a, c, e, g, i) and frequency sweeps (b, d, f, h, j) for 5 wt% C2 (a, b), C6 (c, d), C10 (e, f), C14 (g, h), and C18 (i, j)

Although the inverted vial method is a common method to screen gelation ability, it lacks sensitivity to conclusively determine if the sample is a gel, and it does not provide any information on viscoelastic nature of the sample. Therefore, both oscillatory and frequency sweeps were done to assess the rheological properties of the gels both at 2 and 5 wt% (Figures 4 and 5). As the chain length of the ceramide increased, not only did the elastic modulus (G′) decrease but also the yield point decreased, indicating that an increase in fatty acid chain length decreased the ability of the network to form a continuous 3D coherent network.

Using Clark and Ross‐Murphy's (1987) classical definition of gels, based on the frequency sweeps, at 2 wt%, only C2 in canola oil showed that both G′ and the loss modulus (G″) were frequency independent and hence were true gels. C6 and C10, although they did not flow when the vial was inverted (Figure 2), showed frequency dependence suggesting that the supramolecular structure is an entanglement network (Clark & Ross‐Murphy, 1987). There are many factors which attribute to this decrease, and include differences in solubility, the supramolecular arrangement of the fibers in 3D space, and the crystal–crystal interactions. Beyond C10, the samples were too soft at 2 wt% to transfer from the mold to the rheometer, therefore, no measurements were collected for C14 or C18. At 5 wt% (Figure 5), it is clear that all samples did in fact gel as G′ and G″ were both frequency independent. In addition, as the chain length increased, the elastic moduli decreased as well as the yield stress.

**Figure 6:**
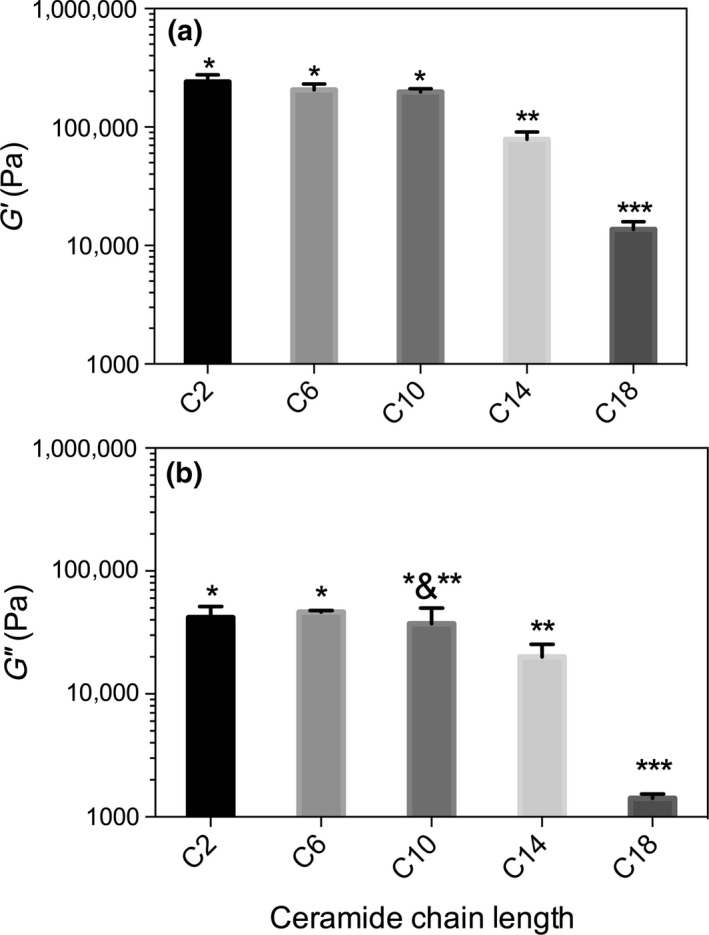
Mean storage (G′) and loss modulus (G″) at 5 wt% obtained from the frequency sweep. Asterisk indicate statistical significance at *p* < .05

**Figure 7:**
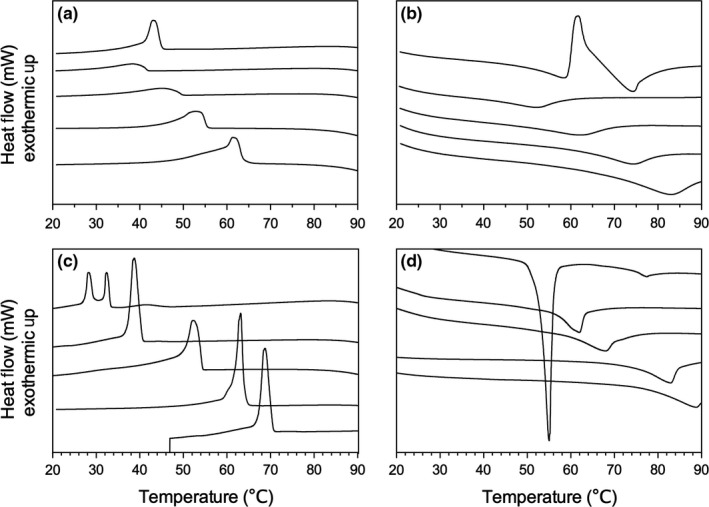
Crystallization (a, c) and melting (b, d) profiles for 2 wt% (a, b) and 5 wt% (c, d) ceramide gels in canola oil. The order from top to bottom for the ceramides is C2, C6, C10, C14, and C18

To aid in the visualization of the differences, G′ and G″ were averaged and the mean and standard deviations were plotted and analyzed using a one‐way ANOVA and a Tukey's post hoc analysis (Figure 6). At 5 wt%, the elastic response for C2, C6, and C10 were statically the same, while C14 and C18 were statically different from the other samples. It is clear from the rheological properties of these gels that there are potential applications for this in edible products. However, it is also important to understand their melting profile to ensure that they remain solid‐like above room temperature. Differential scanning calorimetry was used to observe changes in their melting and crystallization profiles (Figure 7). In general, the transitions between sol and gel follow expected trends, as the chain length increased, both the crystallization and melting temperature increased (Figure 7) and a single peak was observed, with the exception of C2 in canola oil. For C2 in canola oil, it is clear that there are two exothermic events during crystallization and two endothermic transitions during the melting process, this would suggest either a polymorphic solid‐state transition or a liquid crystal transition followed by a transition to a 3D crystalline arrangement. At 2 wt%, the crystallization peak increases to a slightly higher temperature than the C6, which is counter to the global trends observed; this is also reflected in the melting profiles. However, in the melting profile, after an initial melt, there is a recrystallization into a more stable polymorph followed by a second melting peak. When the concentration is increased to 5 wt%, a very weak crystallization peak is observed at ~40°C, followed by another crystallization event, this transition to a much stronger melting peak compared to the other chain length ceramides.

**Figure 8:**
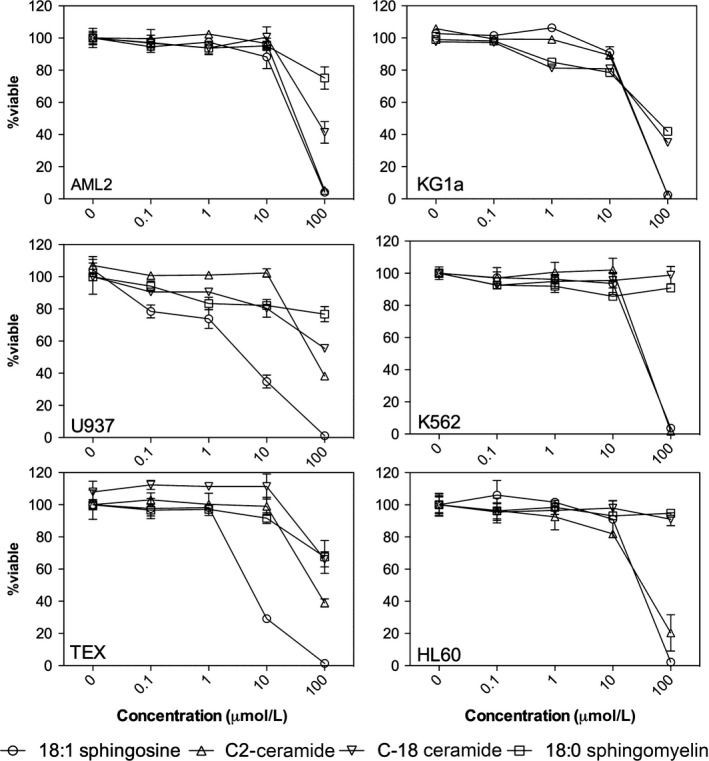
Impact of sphingolipids on leukemia cell viability

**Figure 9:**
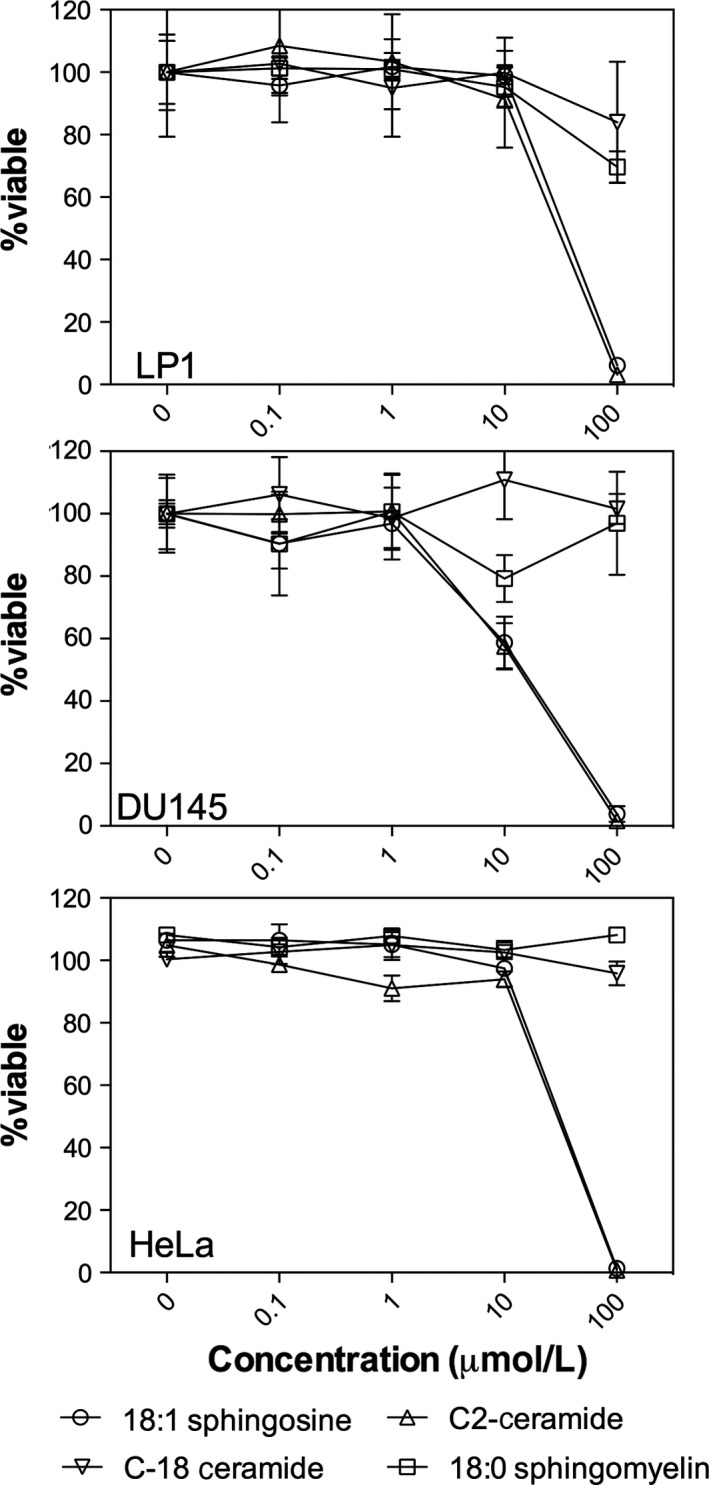
Impact of sphingolipids on LP1 (myeloma), DU145 (prostate), and HeLa (ovarian) cancer cell lines

**Figure 10:**
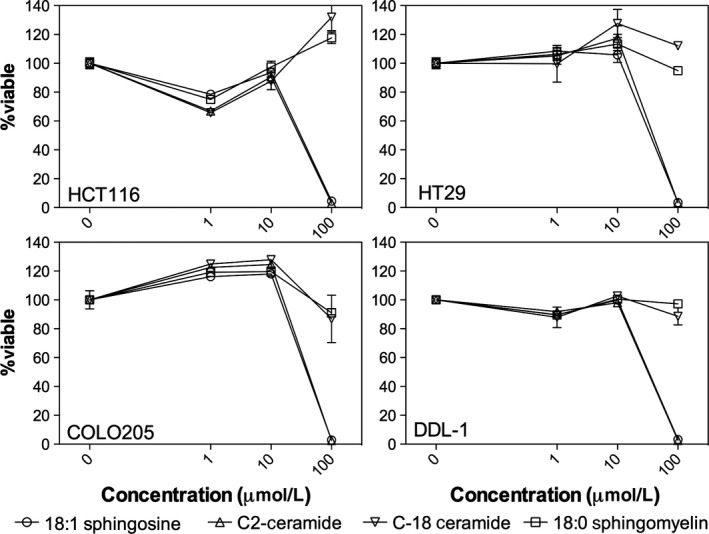
Impact of increasing sphingolipids concentrations on colon cancer cell line viability

It is clearly evident that as the chain length of the ceramide decreases, there is an enhanced ability to self‐assemble into low dimensionality crystals that are more adapt at preventing flow of the continuous oil phase. At this point, it is proof of concept that short‐chain ceramides have tremendous potential to act as a substitute to *trans* and saturated fats in solid fat‐based foods. With this in mind, ceramides have long been reported to have potential as bioactive due to their role in cellular apoptosis (Schmelz & Merrill, 1998b). As such, we compared the ability of C2 and C18 ceramides as well as sphingosine and sphingomyelin to induce cell death in common cancer cell lines. A panel of leukemia cell lines was assessed for their sensitivity to increasing concentrations of ceramides (C2 and C18), sphingomyelin and sphingosine (Figure 8). Across all leukemia cell lines, sphingosine and C2 ceramide displayed the greatest effect on reducing leukemia cell viability. In contrast, C18 ceramide and sphingomyelin had little impact on leukemia cell viability, as measured by the MTS assay. Myeloma, ovarian, and prostate cancer cell lines were also evaluated for their sphingolipid sensitivity (Figure 9). Consistent with the leukemia results, only C2 ceramide and sphingosine reduced cancer cell viability. Finally, sphingolipid bioactivity was tested in a panel of colon cancer cell lines (Figure 10). Similar to the results outlined above, C2 ceramide and sphingosine reduced colon cancer cell viability, whereas C18 ceramide and sphingomyelin had little to no impact on cell viability.

## 4 Conclusion

*N*‐acetyl‐d‐erythro‐sphingosine (C2) is able to convert edible oils into organogels without adding saturated or *trans* fats. As the ceramide fatty acid chain length increases, there is an increase in the melting temperature and a decrease in the elastic modulus of the organogel. C2 ceramide decreases cell viability of colon, prostate, ovarian, and leukemia cell lines, while C18 ceramide does not.

## Conflict of Interest

The authors declare no conflicts of interest.
